# Humoral and Cellular Immune Response in Patients with Hematological Disorders After Three Doses of mRNA COVID-19 Vaccine: A Single-Center Observational Study

**DOI:** 10.3390/vaccines14050369

**Published:** 2026-04-22

**Authors:** Rosa Daffini, Francesco Zecchini, Giulia Venneri, Michele Malagola, Chiara Cattaneo, Stefano Calza, Arnaldo Caruso, Alessandra Tucci, Cinzia Giagulli

**Affiliations:** 1 Hematology Unit, ASST Spedali Civili of Brescia, 25123 Brescia, Italy; rosadaffini@yahoo.it (R.D.); chiara.cattaneo@asst-spedalicivili.it (C.C.); alessandra.tucci@asst-spedalicivili.it (A.T.); 2 Unit of Blood Diseases and Stem Cell Transplantation, ASST Spedali Civili of Brescia, Department of Clinical and Experimental Sciences, University of Brescia, 25123 Brescia, Italy; f.zecchini@studenti.unibs.it (F.Z.); michele.malagola@unibs.it (M.M.); 3 Section of Microbiology, ASST Spedali Civili of Brescia, Department of Molecular and Translational Medicine, University of Brescia, 25123 Brescia, Italy; g.venneri@unibs.it (G.V.); arnaldo.caruso@unibs.it (A.C.); 4 Unit of Biostatistics and Bioinformatics, Department of Molecular and Translational Medicine, University of Brescia, 25123 Brescia, Italy; stefano.calza@unibs.it

**Keywords:** COVID-19, cellular immune response, humoral response, hematological disorders, mRNA vaccine, T cell, anti-CD20, bendamustine, BTK inhibitors, BCL-2 inhibitors

## Abstract

Background: Hematological patients have a high risk of developing severe COVID-19 (37%). Most mRNA vaccine trials in hematological patients showed a low immunogenicity after two doses, while long-term data are scarce. Methods: In this monocentric retrospective observational study, we evaluated humoral and T cell-mediated immune responses in 230 hematological patients after three doses of the Pfizer-BioNTech mRNA COVID-19 vaccine. Patients were stratified by age, disease type/state, prior COVID-19 infection, and treatment status and regimens (anti-CD20 monoclonal antibodies, BTK and BCL-2 inhibitors, and treatment line). Antibody titer to SARS-CoV-2 was assessed by electrochemiluminescence immunoassay and T cell response by QuantiFERON interferon-γ release assay (IGRA). Data were analyzed using univariate (Fisher’s exact test) and Firth’s bias-reduced penalized-likelihood logistic regression. Results: A robust humoral response was observed with 91.55% of patients developing anti-spike antibodies (GMT 988.83 U/mL). Anti-CD20-bendamustine treatment was associated with a significantly lower antibody positivity compared to untreated subjects. Prior COVID-19 infection significantly boosted both antibody positivity (95.9% vs. 85.2%) and GMT (847.02 U/mL vs. 258.79 U/mL). Conversely, T cell response was suboptimal (36.1% positive), particularly in anti-CD20-bendamustine-treated and multi-treated patients (27.1%), but highest in those treated with BTK inhibitors (50%). Multivariable logistic regression analysis linked multiple treatments to lower T cell response. Following vaccination, 29.1% of patients contracted SARS-CoV-2, but only 0.89% developed severe COVID-19. Conclusions: Three doses of mRNA vaccine elicit a strong humoral but a low T cell response, as detected by IGRA, in hematological patients. These findings underscore the importance of completing vaccination before initiating immunosuppressive therapies.

## 1. Introduction

Patients with hematological diseases present an increased risk of developing severe infections with the novel coronavirus SARS-CoV-2 with an adverse clinical outcome [1,2,3,4,5]. Pneumonia and acute respiratory distress syndrome are the main manifestations of the severe form [6], and the mortality rate for COVID-19 among this group of patients is higher than that observed in healthy people [4]. Their increased susceptibility is attributed to both the hematological diseases and the immunosuppressive therapies to which these patients are subjected.

The currently approved Pfizer-BioNTech mRNA COVID-19 vaccine encodes the viral spike protein, the main target of neutralizing antibodies during natural infection. In healthy individuals, vaccination against COVID-19 has been demonstrated to be highly effective in preventing the disease [7,8], and the vaccine response has been widely well-documented in the literature [9,10]. Indeed, clinical studies have demonstrated that a two-dose regimen of the BNT162b2 mRNA COVID-19 vaccine is highly effective in preventing over 90% of symptomatic and severe SARS-CoV-2 infections [7,8,11]. These results are due to the ability of this vaccine to activate both humoral and cell-mediated immunity [12,13].

Initially, hematological patients were excluded from randomized controlled trials due to having a compromised immune system and pharmacological treatments that could interfere with the immune response to vaccines, making it difficult to assess their safety and effectiveness [14]. Subsequently, as the data on COVID-19 vaccine protection were increasing, specific studies were specifically conducted to evaluate the use of these vaccines in this patient population. These studies showed low immunogenicity in COVID-19 vaccines, particularly in individuals with lymphoproliferative disorders and in subjects treated with drugs, including anti-CD20 monoclonal antibodies and Bruton’s tyrosine kinase inhibitors (BTK inhibitors), as well as Janus kinase inhibitors (ruxolitinib) and Bcl-2 inhibitors (venetoclax) [12,13]. While these initial studies clearly demonstrated a generally impaired response in immunocompromised patients compared to healthy individuals, they also highlighted the significant heterogeneity within the hematological patient population itself. This underscored a crucial gap in understanding the specific factors influencing vaccine responses within these diverse subgroups. The prospective study by Maneikis et al. (2021) [12], which analyzed the humoral response in 885 patients with hematological malignancies after vaccination, showed that these subjects, aged between 18 and 60, had a lower anti-S1 IgG antibody response after two doses of the BNT162b2 COVID-19 vaccine compared to healthy subjects. In addition, the lowest antibody levels were observed in patients treated with BTK inhibitors and/or anti-CD20 monoclonal antibodies in the previous 12 months, confirming that these treatments can influence the response of these patients to the vaccine [13].

However, although the dynamics of antibodies are well described [15], there is a paucity of evidence regarding long-term patterns [16], and many studies involve hematological patients only after two doses of mRNA COVID-19 vaccine. Findings from some studies indicate that the development of humoral response does not guarantee long-term protection [17,18]. In particular, Tychala et al. (2021) [19] analyzed the anti-S antibody levels and cell-mediated response of 22 healthcare workers to assess their response following vaccination. The results showed that the participants with high antibody levels had an adequate cellular response, while those with low antibody levels generally had limited or almost no cellular response five months after vaccination [19]. On the other hand, several studies indicate that the presence of memory T cells, obtained through vaccination or as a result of natural infection, is associated with a reduction in the severity of symptoms following a new contact with the virus [16].

For all these reasons, there is a clear need to comprehensively characterize both the humoral and T cell-mediated responses specifically within individuals with compromised immune systems due to hematological disorders and different pharmacological treatments to assess the immunogenicity and efficacy of mRNA-based COVID-19 vaccines.

In this study, we assessed the ability of hematological patients, stratified by age, myeloproliferative or lymphoproliferative disease, disease state (complete/partial remission or onset/stable/progressive), previous SARS-CoV-2 positivity, treatment status (currently on therapy or off therapy) and treatments received, such as monoclonal antibody anti-CD20 alone or in combination with a chemotherapy drug (bendamustine) or BTK or BCL-2 inhibitors, as a first line of treatment or multi-treated, to generate an adequate anti-S antibody and cell-mediated response following three doses of mRNA COVID-19 vaccine.

## 2. Materials and Methods

### 2.1. Patients

From 1 March to 31 July 2022, all patients seen in the Hematology Unit of Brescia Civil Hospital, affected by myeloproliferative and lymphoproliferative diseases, who had received three doses of BNT162b2 mRNA COVID-19 vaccine (Comirnaty; Pfizer Inc., New York, NY, USA and BioNTech SE, Mainz, Germany), or who had received two doses plus a documented COVID-19 infection within the previous two months, were enrolled. This approach reflects the real-world immune landscape of hematological patients during the study period, where a recent natural infection substituted the third dose of vaccine in clinical protocols during the pandemic period, as it was considered an immunological event—in terms of antigenic exposure—equivalent to a booster dose.

Inclusion criteria for the study were as follows: age ≥ 18 years, and a diagnosis of myeloproliferative diseases (such as acute myeloid leukemia, high-risk myelodysplastic syndrome, or primary myelofibrosis) or lymphoproliferative disease (such as myeloma, chronic lymphocytic leukemia, indolent and aggressive B cell non-Hodgkin lymphoma, T cell non-Hodgkin lymphoma, and Hodgkin lymphoma). Most samples were collected during therapy cycles or within 6 months from the end of therapy; only twelve patients were studied at the onset of disease (before starting therapy) and, among this group, five had a therapeutic program with anti-CD20 and/or bendamustine. Regarding anti-CD20 therapies, only 4 patients were evaluated more than 6 months after their last dose.

Exclusion criteria were as follows: autologous or allogeneic hemopoietic stem cell transplant or CAR-T cell treatment in the last year and HIV-1 infection.

This study was approved on 28 November 2025, by the Territorial Ethics Committee Lombardy 6 (trial registration NP6610), with headquarters at IRCSS Foundation Saint Matthias Polyclinic in Pavia, and all the patients signed the informed consent.

### 2.2. Humoral Response

Serum samples were analyzed for total antibody titers (IgA, IgM, and IgG) against the SARS-CoV-2 nucleocapsid (N) and spike (S) proteins. The quantitative determination was performed using the Elecsys^®^ anti-SARS-CoV-2 electrochemiluminescence immunoassays (ECLIA) on the Roche Cobas platform (Roche Diagnostics International Ltd., Rotkreuz, Switzerland; software version 06.10), as previously described [20] and following the manufacturer’s instructions. Anti-N antibodies (positivity threshold ≥1.0 U/mL) were systematically tested at enrollment for all participants to stratify the cohort. This allowed us an accurate identification of patients with prior natural infection, ensuring that subjects with undocumented or asymptomatic SARS-CoV-2 infections were correctly identified and stratified for analysis. Only cases with complete datasets were evaluated. Anti-S antibody levels were expressed in U/mL. Notably, the Elecsys^®^ anti-S results are calibrated against the First WHO International Standard (NIBSC code 20/136), with a conversion factor, where 1 U/mL is approximately 1.02 BAU/mL [21]; thus, U/mL and BAU/mL values are considered equivalent for this analysis. Samples were classified as reactive (positive) if the concentration was ≥0.80 U/mL for anti-S antibodies, while lower values were defined as non-reactive (negative).

### 2.3. T Cell Response

The cellular immune response was evaluated using the QuantiFERON (QFN) SARS-CoV-2 interferon-γ release assay (IGRA) (Qiagen, Hilden, Germany), as previously described [20]. This method quantifies the IFN-γ produced by CD4^+^ or CD4^+^ and CD8^+^ lymphocytes by ELISA after in vitro stimulation of heparinized whole blood. The system employs specific SARS-CoV-2 antigens: the Ag1 tube contains CD4^+^ epitopes from the S1 subunit, which includes the Receptor Binding Domain (RBD), while the Ag2 tube includes both CD4^+^ and CD8^+^ epitopes from the S1 and S2 subunits. The use of the S1 subunit minimizes potential cross-reactivity with other human coronaviruses, as the S2 region exhibits higher conservation across the Coronaviridae family [22].

In addition, since QuantiFERON may exhibit limitations in sensitivity and specificity, when we assessed cellular immunity in immunocompromised individuals, due to inherent immune dysfunction or anergy associated with their condition or therapies, a control tube set was employed. This set, including negative (Nil) and positive (Mitogen) control tubes, was crucial to exclude non-specific background reactivity and to filter out individuals with a global T cell anergy, respectively, ensuring the reliability of the results. Only QuantiFERON results with a positive mitogen control (indicating preserved general T cell responsiveness) were included in the statistical analysis. Patients whose mitogen tube did not yield a positive response were classified as “indeterminate” and were explicitly excluded from the immunological data analysis.

Briefly, heparinized blood tubes were incubated at 37 °C for 16 h and then centrifuged for 15 min at 2500× *g*. The recovered plasma was analyzed for IFN-γ using the automated DYNEX DS2 ELISA system (Dynex Technologies, Chantilly, VA, USA) in compliance with the manufacturer’s guidelines. To ensure specificity, final IFN-γ concentrations (IU/mL) were calculated by subtracting the background (Nil) values. A threshold of ≥0.15 IU/mL was utilized to define a positive T cell response.

### 2.4. Study Endpoints

The primary endpoint of this study was to analyze the rate of humoral and cellular response after the third dose of mRNA COVID-19 vaccine in different subgroups of hematological patients by comparing their immunological response. In particular, the subcategories analyzed were patients older or younger than 65 years, patients with lymphoproliferative vs. myeloproliferative diseases, patients at their first line of treatment vs. multi-treated, and patients with or without a previous SARS-CoV-2 infection. Concerning treatments, the focus was to evaluate the effect of different deep immunosuppressive therapies, like anti-CD20 monoclonal antibodies (rituximab and obinutuzumab), a chemotherapy drug (bendamustine), BTK inhibitors (ibrutinib, acalabrutinib), and BCL-2 inhibitors (venetoclax) with respect to mRNA COVID-19 vaccination.

Secondary endpoints of this study were the evaluation of the overall survival and incidence of COVID-19 disease, according to the immunological status of different patients, with a follow-up of 9–14 months from the collection of the samples.

### 2.5. Statistical Analysis

Correlations with clinical and therapeutic features were studied by a univariate analysis (Fisher’s exact test).

To identify demographic and clinical factors independently associated with post-vaccination humoral and cellular immune responses, Firth’s bias-reduced penalized-likelihood logistic regression was performed. The dependent (outcome) variables considered were as follows: (1) anti-S antibody positivity (defined as a titer ≥ 0.80 U/mL) and (2) QuantiFERON test positivity (defined as IFN-γ ≥ 0.15 IU/mL). In the multivariable logistic regression analysis, the following independent variables, considered clinically relevant and potential predictors of immune response, were included: age (<65 vs. ≥65 years), type of hematological diagnosis (myeloproliferative vs. lymphoproliferative disease), disease state (complete/partial remission vs. onset/stable/progressive), previous COVID-19 infection (Yes/No), treatment status (currently on therapy vs. off therapy), treatment line and specific immunosuppressive therapies (anti-CD20 therapy (Yes/No), bendamustine (Yes/No), anti-CD20 with bendamustine (Yes/No), BTK inhibitors (Yes/No), Bcl-2 inhibitors (Yes/No), and first line of treatment vs. multi-treated. Model calibration was checked both graphically and by estimating the intercept/slope of the calibration plot: in all models both intercept and slope were coherent (intercept = 0, slope = 1). Variance inflation factors were also computed and were all below 2. Results are presented as odds ratios (ORs) with their respective 95% confidence intervals (95% CIs). An odds ratio > 1 indicates a higher probability of a positive outcome, while an OR < 1 indicates a lower probability.

The results were considered statistically significant if the *p*-value was ≤0.05. All statistical analyses were performed using software R, version 4.5.0 (https://www.r-project.org/ (accessed on 7 January 2026).

## 3. Results

According to the inclusion and exclusion criteria, 230 patients were consecutively enrolled. All their demographic and clinical characteristics are reported in Table 1.

At the time of assessment, 76 patients (33.04%) were at the beginning of their treatment program, 75 (32.60%) were in partial remission, 51 (22.2%) had a documented complete remission, 21 (9.13%) had a progressive disease, and 7 (3.04%) had stable disease. The treatment details are described in Table 2.

Both humoral and T cell responses were assessed at a median time of 4 months after the last dose of vaccine, at a median time of 2 months from the beginning of the treatment, and less than 1 month from the last therapy administration.

The humoral response was present in 209 patients (91.55%) with a geometric mean titer (GMT) of 988.83 U/mL (range < 0.4–5000 U/mL). The T cell-mediated response, assessed by the QuantiFERON SARS-CoV-2 assay, was positive in 72 patients (35.3%) and negative in 132 (64.07%). Only 26 patients were indeterminate, since they did not respond to positive mitogen control, showing a global T cell anergy and, consequently, they were excluded from the analyses. However, among these 26 patients, 22 showed a humoral response, while four did not produce anti-spike antibodies. Among all patients enrolled (230), 67 (29.13%) were reactive for both humoral and T cell response, 121 (52.61%) showed only humoral response, and five showed only T cell response (2.17%), while 11 (4.78%) resulted negative for both humoral and cellular response.

In Table 3, we show a comparative analysis of humoral response among the different subgroups of patients based on demographic and clinical characteristics. The analysis shows that mRNA COVID-19 vaccination is able to generate an antibody response in more than 80% of patients (range 80–100%), independently from age (<65 or >65 years), type of hematological disease (myeloproliferative or lymphoproliferative diseases), disease state (complete/partial remission or onset/stable/progressive), history of previous COVID-19 infection, or treatment status (on or off therapy).

Patients with previous COVID-19 infection showed significantly higher anti-S antibody positivity (95.9% vs. 85.2%; *p* = 0.006) and significantly higher anti-S antibody GMTs (847.02 U/mL vs. 258.79 U/mL; *p* = 0.005) compared to those without prior infection, indicating that a prior infection status strongly influences antibody presence (Table 3). Significant differences were found in anti-S antibody GMTs based on the type of hematological disease (myeloproliferative vs. lymphoproliferative disease; *p* = 0.003) and disease state (CR/PR vs. O/SD/PD; *p* = 0.03).

In addition, the multivariable logistic regression analysis established that a history of myeloproliferative disease (vs. lymphoproliferative disease) and a previous COVID-19 infection (vs. no previous COVID-19) represent significant independent predictors of anti-S antibody positivity, with ORs of 0.11 (95% Cis: 0.001, 0.83; *p* = 0.027) and 3.87 (95% CIs: 1.49, 11.7; *p* = 0.005) (Table 4).

In Table 5, we show a comparative analysis of the humoral response among the different subgroups of patients treated with specific immunosuppressive therapies, such as anti-CD20 with or without bendamustine, BTK inhibitors, and Bcl-2 inhibitors (venetoclax) and the treatment line (first line of treatment or multi-treated). All patients affected by myeloproliferative diseases (100%) or treated with BCL-2 inhibitors (venetoclax) (100%) were able to generate anti-spike antibodies after vaccination. On the other hand, patients treated with anti-CD20 antibodies in association with bendamustine or treated with BTK inhibitors had the lowest seropositivity rate (81% and 80%, respectively). Then, a significant difference in anti-S antibody positivity was observed between patients treated with anti-CD20-bendamustine therapy and those who were not treated (*p* = 0.03); the treated group exhibited significantly lower anti-S antibody GMTs (350.14 U/mL vs. 964.22 U/mL, *p* = 0.016) than the untreated one (Table 5). A statistically significant difference in GMTs was also observed between the two groups of patients treated or not treated with venetoclax (2905.70 vs. 688.32, respectively, *p* = 0.02) (Table 5).

To determine the independent effect of these specific treatments on anti-S antibody positivity, a multivariable logistic regression analysis was performed (Table 6). Therapy with anti-CD20-bendamustine was independently associated with a significantly lower likelihood of anti-S antibody positivity, with an odds ratio (OR) of 0.30 (95% CI: 0.08, 1.01; *p* = 0.05).

Then, we evaluated the cellular immune response using the QuantiFERON test in different subgroups of patients stratified by demographic and clinical characteristics (Table 7). In the univariate analysis, no significant differences were observed in the percentage of QuantiFERON-positive patients stratified by age, type of hematological disease, disease state, history of COVID-19 infection, and therapy status. On the other hand, the analysis of mean IFN-γ levels in response to specific antigens (Ag1 and Ag2) revealed some significant differences. Patients with myeloproliferative diseases showed significantly lower mean Ag1 levels (0.17) compared to those with lymphoproliferative diseases (0.30; *p* = 0.015). Additionally, patients with previous COVID-19 infection had significantly higher mean Ag2 levels (0.46) compared to those without infection (0.30; *p* = 0.038). No other significant differences in mean Ag1 or Ag2 levels were observed across the other stratified groups (age, previous COVID-19, therapy status).

To identify independent factors associated with a positive QuantiFERON test, a multivariable logistic regression analysis was performed (Table 8). No demographic or clinical characteristic emerged as a significant independent predictor of T cell response.

The impact of specific treatment regimens on the cellular immune response using the QuantiFERON test was further assessed. In Table 9, we show that a very low percentage of treated subjects (from 27.1 to 50%) responded positively to the QuantiFERON SARS-CoV-2 test. In detail, the lowest percentage of positive patients for the QuantiFERON test was observed in patients treated with anti-CD20–bendamustine and multi-treated subjects (27.1%), while the highest percentage was detected in patients treated with BTK inhibitors (50%). In the univariate analysis, no significant differences were observed in the percentage of QuantiFERON-positive patients across the different treatment groups (anti-CD20 therapy, anti-CD20-bendamustine combination therapy, BTK inhibitor use, venetoclax therapy, and treatment line). However, analysis of mean IFN-γ levels in response to specific antigens, Ag1 and Ag2, revealed some significant differences (Table 9). Patients not treated with anti-CD20-bendamustine showed significantly higher mean Ag2 levels (0.52) compared to those treated (0.13, *p* = 0.031); on the other hand, multi-treated patients exhibited a significantly lower mean Ag2 level (0.22) compared to those on first-line treatment (0.52; *p* = 0.02). No other significant differences in mean Ag1 or Ag2 levels were observed across the remaining stratified groups based on specific therapies.

To determine the independent effect of specific treatments on T cell response, a multivariable logistic regression analysis was performed (Table 10). The analysis indicated that multiple treatments were independently associated with a significantly lower likelihood of a positive QuantiFERON test, with an odds ratio (OR) of 0.49 (95% CI: 0.23, 0.98; *p* = 0.044). No significant independent association with T cell response was found for the other specific treatments.

Concerning the evaluation of the overall survival and incidence of COVID-19 disease, our finding indicated that, after three doses of the COVID-19 vaccine, 67 patients (29.1%) experienced a SARS-CoV-2 infection: in 53 (79%), the symptoms were mild or absent, while hospitalization was necessary in 14 (21%). Among the hospitalized patients, only two, who were treated with anti-CD20 in the last six months, contracted severe COVID-19 disease (0.89% of the total individuals analyzed in this study). One of them was negative for both humoral and cellular immune response; the other one had a positive humoral response with a very low titer (10.7 U/mL) and was unreactive for T cell response.

Among the 36 patients who experienced progressive disease (16.6%), seven (3%) documented a SARS-CoV-2 infection after the third dose of vaccine. In only one case, COVID-19 disease caused a delay in the beginning of treatment.

## 4. Discussion

Discordant data have been reported so far regarding humoral and cellular response to mRNA COVID-19 vaccine in immunocompromised patients. In this observational monocentric study, involving subjects with different hematological diseases, we found a surprising optimal humoral response with 91.55% of patients developing anti-spike antibodies and a geometric mean titer of 988.83 U/mL, but a suboptimal T cell response with a very low percentage of subjects (from 27.1 to 50%) who responded positively to the QuantiFERON SARS-CoV-2 assay. These data are in contrast with some previously published studies, which highlighted the detrimental effect of anti-B-cell therapies and the negative impact of immunosuppressive drugs on humoral response induced by the vaccine, and, on the other hand, the good capability of hematological patients to develop a robust T cell response, despite the absence of humoral response [19,20,22,23,24,25]. Several factors could be responsible for these discrepancies. Specifically, while previous studies often evaluated humoral response after two vaccine doses or only a few days/weeks after the third dose, focusing on the peak immunogenicity window (2–4 weeks), our observed results should be interpreted considering our longer follow-up interval (median 4 months) post-third dose. Although this timeframe might introduce a survivor bias or reflect delayed humoral response—common factors in hematological patients—it offers a perspective on the durability and persistence of the vaccine response beyond the initial peak. In these fragile hosts, understanding the maintenance of antibody levels is as clinically relevant as the initial peak, as it defines the window of protection during ongoing waves of infection. Thus, our findings suggest that hematological patients who successfully seroconvert can maintain stable antibody levels for several months, providing insights into the sustained protection achievable in this vulnerable population. However, Nguyen et al. (2023) obtained data consistent with our results, observing a humoral response in 87% of their patients with lymphoproliferative diseases after the third dose of mRNA COVID-19 vaccine [26].

Furthermore, most of the previous studies analyzed humoral and T cell response in patients who received the vaccine while they were undergoing treatment, while we assessed patients who had completed the vaccination course with three doses before starting therapy or after its conclusion. This aspect is important because it is known that an active treatment during the vaccine course, particularly with lymphodepleting therapies, can have a negative impact on humoral response [27,28].

Moreover, many of these studies included only patients with lymphoproliferative diseases, which are known to have an unfavorable immunological status both for the hematological disease and treatment received [29]. In our study, we considered both lymphoproliferative and myeloproliferative diseases and observed different seropositivity rates, equal to 89.4% and 100%, respectively.

In addition, patients with ongoing treatment, based on anti-CD20-bendamustine showed a significantly lower anti-S antibody positivity with respect to subjects who did not receive the treatment (Table 5), and multivariate logistic regression further confirmed this as an independent association (Table 6). This data suggests that this kind of treatment might impact the development or maintenance of anti-spike antibodies, potentially impacting vaccine response. The detrimental effect of anti-CD20 antibodies on the humoral response to the anti-SARS-CoV-2 vaccination is widely supported by many studies in the literature, including recent findings in both hematological and non-hematological inflammatory conditions [30,31].

Unlike other studies that often exclude patients with prior SARS-CoV-2 infection, we included them in our analysis. We observed a significant difference between patients with or without a previous documented COVID-19 infection; this aspect could contribute to increasing the percentage of our patients affected by lymphoproliferative diseases able to mount a good humoral immune response. Nguyen et al. (2023) found that hematological patients with COVID-19 breakthrough infections generate higher antibody responses in comparison to subjects who have never been infected [26].

However, in our study we have not analyzed the neutralizing activity of anti-spike antibodies. We acknowledge that the 0.80 U/mL threshold is a technical cutoff for seropositivity and does not necessarily imply a correlate of protection. Neutralization assays are crucial in providing a functional measure of antibody-mediated protection against COVID-19, although the relationship between in vitro neutralization levels and protection against symptomatic COVID-19 has been widely described [32]. In this context, while binding antibody titers measured by the Roche ECLIA assay have been shown to correlate strongly with neutralizing activity in several cohorts [33], the absence of functional assays and the use of a low positivity threshold may lead to an overestimation of protective immunity. So, our findings on anti-S antibody levels provide valuable insights into the magnitude of the humoral response, reflecting primarily antibody presence and binding capacity rather than their direct functional ability to neutralize the virus. However, the very low rate of severe COVID-19 (0.89%), observed in our cohort, is noteworthy when compared with the 30-day mortality rate of 39.2% reported in patients from the same hematology department at the beginning of the pandemic [34]. Although this comparison is purely descriptive and must be interpreted with caution due to different viral variants and improved therapeutic strategies, the high seropositivity rate observed after three doses aligns with a more favorable clinical trend in this fragile population. Future studies including functional neutralization assays could offer a more complete picture of antibody-induced protection against COVID-19.

Regarding the role of T cell response, a protective function against severe COVID-19 disease in hematological patients was reported [35]. Corradini et al. (2023) demonstrated that a T cell-mediated response was present in most patients after two doses of vaccine, even in the absence of seropositivity, and that this response was independent of treatment, with a significant increase observed 2–4 weeks following the third dose [25]. In contrast, Shroff et al. (2021) reported no T cell response improvement one week after the third dose [36].

In our study, the analysis of QuantiFERON data, at a median time of 4 months after the third dose of vaccine, showed a low T cell response, despite the presence of humoral immunity. In particular, the T cell immune response rate was lower in multi-treated patients (27.1%) compared to those treated with BTK inhibitors (50%), suggesting that different treatment strategies may have a negative or positive impact on their capability to develop a T cell-mediated response to COVID-19 vaccination and, consequently, to SARS-CoV-2 infection. Notably, the relatively higher T cell response observed in patients treated with BTK inhibitors is an intriguing finding that warrants further mechanistic exploration in future studies. The low T cell response rate observed in these highly vulnerable subgroups raises important concerns about the durability of cellular immunity in this population and highlights a potential ongoing need for complementary protective strategies, such as prophylactic monoclonal antibodies or additional vaccine boosters. Taken together, all these observations suggest the possibility of different results regarding T cell responses based on different time points set in clinical trials and a rapid decline in T cell response in immunocompromised patients after 4 months from the third dose of vaccine.

The extreme variability of the results obtained in these highly immunocompromised patients, where many variables interfere with the humoral and T cell response, in particular disease-intrinsic immunodeficiency and type and timing of treatment, underscores the need for tailored vaccination schedules.

Furthermore, we acknowledge the intrinsic limitations of the QuantiFERON SARS-CoV-2 assay in the setting of hematological malignancies, since it depends on the presence of a sufficient number of functional T cells and its reduced sensitivity can lead to indeterminate results. In our study, the exclusion of 26 patients (11.3%), due to indeterminate responses to the mitogen control, was necessary for statistical consistency, although these cases might represent the most immunosuppressed subgroup and their removal might lead to an overestimation of the overall cellular immune competence of the cohort.

To overcome these limitations, future investigations could carry out more in-depth analyses, performed by tetramer staining or intracellular cytokine staining (ICS) by flow cytometry, to provide deeper insights into specific T cell subsets and epitope-specific responses, even in paucicellular samples where IGRA might fail. However, such approaches currently remain less standardized for large-scale clinical monitoring compared to the QuantiFERON assay.

A possible confounding factor, not analyzed in our study, is the influence of the different genetic characteristics of the subjects. Recent observations indicate that specific HLA class I and class II antigens, identified through next-generation sequencing, seem to be associated with a varying severity of SARS-CoV-2 infection [37]. Furthermore, different mutations in ACE2, a key target for viral spike protein, have been reported to correlate with a variable vulnerability to COVID-19 infection in patients with solid cancers due to an altered ligand–receptor affinity [38,39].

A limitation of this study is its monocentric nature. However, our patient characterization and statistical analyses are aimed at mitigating these effects and enhancing the generalizability of our findings to real-world clinical practice.

## 5. Conclusions

In conclusion, the high rate of seropositive patients in our cohort of hematological subjects highlights the importance of a third dose of vaccine and underlines the need to complete the vaccination cycle before starting treatment. However, only further longitudinal prospective studies with larger sample sizes and patient cohorts from different geographical locations will allow us to clarify the discordant data on humoral and cellular response obtained in different studies involving immunocompromised vulnerable patients.

## Figures and Tables

**Table 1:** Demographic and clinical characteristics of patients enrolled in the study. The table shows the distribution of patients by sex, age, type of hematological malignancies, clinical status, and line of treatment. For each category, the number of patients and the percentage are reported. AML, acute myeloid leukemia; MDS, myelodysplastic syndrome; PMF, primary myelofibrosis; CLL, chronic lymphocytic leukemia; SLL, small lymphocytic lymphoma; NHL, non-Hodgkin lymphoma; HL, Hodgkin lymphoma.

Demographic and Clinical Characteristics		
	*n*°	%
**Sex**		
Male	137	57.4%
Female	93	42.6%
**Age**		
Median	71	
Range	23–92	
**H** **ematological Malignancies**		
AML, MDS, PMF	32	13.9%
CLL/SLL and Indolent B Cell NHL	83	36.1%
Aggressive B Cell NHL	60	26.1%
Hodgkin Lymphoma (HL)	20	8.6%
T cell NHL	6	2.6%
Multiple Myeloma (MM)	29	12.6%
**Clinical Status**		
Complete Remission (CR)	51	22.2%
Partial Remission (PR)	75	32.6%
Stable Disease (SD)	7	3.1%
Progressive Disease (PD)	21	9.1%
Onset (O)	76	33%
**Line of Treatment**		
1	160	69.6%
2+	70	30.4%

**Table 2:** The table describes the types of therapies received by patients and shows the number and percentage of patients for each therapy type. * pembrolizumab, brentuximab, ** mogamulizumab, pembrolizumab/nivolumab, daratumumab (±lenalidomide and/or proteasoma inhibitors), *** gilteritinib, ruxolitinib, fedratinib. Venetoclax was not used as monotherapy, but patients received this drug in combination with azacitidine or ibrutinib, so they were included in the chemotherapy or BTK inhibitor subgroups.

Therapy	*n*° (%)
Chemotherapy	50 (21.7%)
AntiCD20 + Chemotherapy	104 (45.2%)
AntiCD20 + Bispecific Antibodies	11 (4.8%)
Immuno-Chemotherapy (no anti-CD20) *	2 (0.8%)
Immunotherapy **	38 (16.5%)
BTK Inhibitors	18 (7.8%)
Target Therapy ***	4 (1.7%)
Follow-up	3 (1.3%)

**Table 3:** Anti-S antibody response and geometric mean titers (GMTs) of anti-S antibodies, after three doses of mRNA COVID-19 vaccine, in hematological patients stratified by various demographic and clinical characteristics (age, type of disease, disease status, history of COVID-19 infection, and treatment status). Abs, antibodies; GMT, geometric mean titer; anti-S, anti-spike; CI, confidence interval; CR, complete remission; PR, partial remission; O, onset; SD, stable disease; PD, progressive disease; Pts, patients.

	Abs Anti-Spike		Tot Pts	*p*-Value	GMT of Anti-S Antibody (U/mL)	Ratio	95% CI	*p*-Value
	Positive	Negative						
≤65 years	78 (94%)	5 (6%)	83	0.2	625.07 (326.61, 1196.25)	0.67	0.31, 1.51	0.3
>65 years	131 (89.1%)	16 (10.9%)	147		420.8 (258.37, 685.32)			
Myelo proliferative diseases	32 (100%)	0 (0%)	32	0.051	2099.96 (751.93, 5864.68)	0.18	0.06, 0.55	0.003
Lympho proliferative diseases	177 (89.4%)	21 (10.6%)	198		383.07 (253.49, 578.88)			
CR/PR	112 (88.9%)	14 (11.1%)	126	0.4	292.22 (173.97, 490.84)	3.07	1.43, 6.62	0.003
O/SD/PD	97 (93.3%)	7 (6.7%)	104		897.60 (507.19, 1588.53)			
Previous COVID-19	117 (95.9%)	5 (4.1%)	122	0.006	847.02 (500.61, 1433.11)	3.27	1.53, 7.02	0.005
No previous COVID-19	92 (85.2%)	16 (14.8%)	108		258.79 (147.98, 452.56)			
On therapy	163 (90.6%)	17 (9.4%)	180	>0.9	413.8 (266.66, 642.12)	0.48	0.19, 1.23	0.13
Off therapy	46 (92%)	4 (8%)	50		862.1 (374.53, 1984.38)			

**Table 4:** This table presents the results of a Firth’s bias-reduced penalized-likelihood logistic regression analysis, assessing different demographic and clinical factors associated with anti-S antibody positivity after three doses of mRNA COVID-19 vaccine in hematological patients. The table shows the odds ratios (ORs), 95% confidence intervals (CIs), and *p*-values for each covariate. CI, confidence interval; CR, complete remission; PR, partial remission; O, onset; SD, stable disease; PD, progressive disease.

	OR	95% CI	*p*-Value
≤65 years	0.54	0.18, 1.46	0.2
>65 years			
Myeloproliferative diseases	0.11	0.001, 0.83	0.027
Lymphoproliferative diseases			
CR/PR	1.82	0.69, 5.23	0.2
O/SD/PD			
Previous COVID-19	3.87	1.49, 11.7	0.005
No previous COVID-19			
On therapy	1.17	0.32, 3.68	0.8
Off therapy			

**Table 5:** Anti-S antibody response and geometric mean titers (GMTs) of anti-S antibody, after three doses of mRNA COVID-19 vaccine, in hematological patients stratified by various treatment regimens (anti-CD20 with or without bendamustine, BTK inhibitors, or Bcl-2 inhibitor venetoclax) and treatment line. * rituximab or obinutuzumab. Pts = patients.

	Ab Anti-Spike		Tot Pts	*p*-Value	GMT of Anti-S Antibody (U/mL)	Ratio	95% CI	*p*-Value
	Positive	Negative						
Anti-CD20 *	77 (86.5%)	12 (13.5%)	89	0.078	571.99 (348.91, 937.71)	0.56	0.28, 1.10	0.093
No anti-CD20	86 (94.5%)	5 (6.5%)	91		1024.52 (641.58, 1636.03)			
Anti-CD20 *-bendamustine	34 (81%)	8 (19%)	42	0.03	350.14 (168.52, 727.49)	0.36	0.16, 0.82	0.016
No anti-CD20-bendamustine	129 (93.5%)	9 (6.5%)	138		964.22 (659.77, 1409.18)			
BTK inhibitor	12 (80%)	3 (20%)	15	0.2	1057.28 (312.02, 3582.59)	1.40	0.40, 4.93	0.6
No BTK inhibitor	151 (91.5%)	14 (8.5%)	165		757.63 (530.22, 1082.57)			
Bcl-2 inhibitor (venetoclax)	14 (100%)	0 (0%)	14	0.4	2905.70 (913.16, 9245.96)	4.22	1.27, 14.0	0.020
No Bcl-2 inhibitor (venetoclax)	149 (89.8%)	17 (10.2%)	166		688.32 (483.86, 979.17)			
First line of treatment	108 (91.5%)	10 (8.5%)	118	0.6	845.18 (555.71, 1285.42)	0.78	0.38, 1.60	0.5
Multi-treated	55 (88.7%)	7 (11.3%)	62		658.68 (364.04, 1191.79)			

**Table 6:** Firth’s bias-reduced penalized-likelihood logistic regression analysis of factors associated with anti-S antibody positivity, focusing on specific treatment lines and regimens in hematological patients after three doses of mRNA COVID-19 vaccine. This table shows the odds ratios (ORs), 95% confidence intervals (CIs), and *p*-values for each covariate. * rituximab or obinutuzumab.

	OR	95% CI	*p*-Value
Anti-CD20 *	0.543	0.14, 1.87	0.3
No Anti-CD20			
Anti-CD20 *-bendamustine	0.30	0.08, 1.01	0.05
No anti-CD20-bendamustine			
BTK inhibitor	0.22	0.054, 1.03	0.055
No BTK inhibitor			
Bcl-2 inhibitor (venetoclax)	1.73	0.16, 237	0.7
No Bcl-2 inhibitor (venetoclax)			
First line of treatment	0.55	0.18, 1.73	0.3
Multi-treated			

**Table 7:** Cellular immune response and Ag1- and Ag2-stimulated IFN-γ levels in hematological patients following three doses of mRNA COVID-19 vaccine. This table presents the percentage of patients with a positive QuantiFERON test and the mean levels of IFN-γ in response to SARS-CoV-2 antigens (Ag1 and Ag2), stratified by various demographic and clinical characteristics (age, type of hematological disease, disease state, history of COVID-19 infection, and therapy status). Std dev, Standard Deviation; Ag1, Antigen 1; Ag2, Antigen 2; CR, complete remission; PR, partial remission; O, onset; SD, stable disease; PD, progressive disease; Pts, patients. ^1^ Fisher’s exact test, ^2^ Wilcoxon rank sum test.

	QuantiFERON		Tot Pts	*p* ^1^	QuantiFERON		QuantiFERON	
	Positive	Negative			Mean (Std Dev) Ag1 (IU/mL)	*p* ^2^	Mean (Std Dev) Ag2 (IU/mL)	*p* ^2^
≤65 years	27 (38.6%)	43 (61.4%)	70	0.5	0.23 (0.63)	0.7	0.31 (0.79)	0.6
>65 years	45 (33.6%)	89 (66.4%)	134		0.31 (1.17)		0.43 (1.52)	
Myeloproliferative diseases	10 (35.7%)	18 (64.3%)	28	>0.9	0.17 (0.55)	0.015	1.01 (2.68)	0.7
Lymphoproliferative diseases	62 (35.2%)	114 (64.8%)	176		0.30 (1.07)		0.29 (0.88)	
CR/PR	37 (33.6%)	73 (66.4%)	110	0.7	0.21 (0.79)	0.5	0.34 (1.30)	0.4
O/SD/PD	35 (37.2%)	59 (62.8%)	94		0.37 (1.22)		0.44 (1.40)	
Previous COVID-19	42 (38.5%)	67 (61.5%)	109	0.3	0.31 (0.99)	0.5	0.46 (1.35)	0.038
No previous COVID-19	30 (31.6%)	65 (68.4%)	95		0.25 (1.03)		0.30 (1.25)	
On therapy	68 (35%)	126 (65%)	194	0.7	0.31 (1.12)	0.8	0.37 (1.33)	0.2
Off therapy	4 (40%)	6 (60%)	10		0.20 (0.39)		0.45 (1.22)	

**Table 8:** Firth’s bias-reduced penalized-likelihood logistic regression analysis of factors associated with T cell response in hematological patients after three doses of mRNA COVID-19 vaccine. This table presents the results of a multivariable logistic regression analysis. Odds ratios (ORs) with corresponding 95% confidence intervals (CIs) and *p*-values are shown for demographic and clinical characteristics associated with a positive QuantiFERON test. CR, complete remission; PR, partial remission; O, onset; SD, stable disease; PD, progressive disease.

	OR	95% CI	*p*-Value
≤65 years	0.84	0.46, 1.54	0.6
>65 years			
Myeloproliferative diseases	0.93	0.41, 2.17	0.9
Lymphoproliferative diseases			
CR/PR	1.14	0.62, 2.08	0.7
O/SD/PD			
Previous COVID-19	1.34	0.75, 2.39	0.3
No previous COVID-19			
On therapy	0.78	0.22, 2.99	0.7
Off therapy			

**Table 9:** Cellular immune response and antigen-stimulated IFN-γ levels after three doses of mRNA COVID-19 vaccine in hematological patients stratified by treatment received. This table presents the percentage of patients with positive QuantiFERON tests and the mean levels of IFN-γ in response to SARS-CoV-2 antigens (Ag1 and Ag2), stratified by various treatment regimens. Std Dev, standard deviation; Ag1, Antigen 1; Ag2, Antigen 2; Pts, patients. ^1^ Fisher’s exact test, ^2^ Wilcoxon rank sum test. * rituximab or obinutuzumab.

	QuantiFERON		Tot Pts	*p* ^1^	QuantiFERON		QuantiFERON	
	Positive	Negative			Mean (Std Dev) Ag1 (IU/mL)	*p* ^2^	Mean (Std Dev) Ag2 (IU/mL)	*p* ^2^
Anti-CD20 *	32 (32.3%)	67 (67.7%)	99	0.5	0.35 (1.32)	0.8	0.26 (0.92)	0.5
No Anti-CD20	36 (37.9%)	59 (62.1%)	95		0.29 (0.78)		0.60 (1.72)	
Anti-CD20 *— bendamustine	13 (27.1%)	35 (72.9%)	48	0.2	0.32 (1.45)	0.2	0.13 (0.28)	0.031
No anti-CD20— bendamustine	55 (37.7%)	91 (62.3%)	146		0.32 (0.95)		0.52 (1.57)	
BTK inhibitor	9 (50%)	9 (50%)	18	0.2	0.24 (0.39)	0.2	0.27 (0.52)	0.6
No BTK inhibitor	59 (34%)	118 (67%)	176		0.33 (1.14)		0.44 (1.44)	
Bcl-2 inhibitor (venetoclax)	7 (43.8%)	9 (56.2%)	16	0.6	0.24 (0.74)	0.14	1.78 (3.65)	0.5
No Bcl-2 inhibitor (Venetoclax)	61 (34%)	118 (66%)	178		0.33 (1.09)		0.31 (0.87)	
First line of treatment	52 (38.5%)	83 (61.5%)	135	0.14	0.37 (1.24)	0.2	0.52 (1.59)	0.02
Multi-treated	16 (27.1%)	43 (72.9%)	59		0.52 (1.59)		0.22 (0.62)	

**Table 10:** Firth’s bias-reduced penalized-likelihood logistic regression of factors associated with T cell response, focusing on specific treatment regimens, in hematological patients after three doses of mRNA COVID-19 vaccine. This table presents the results of a multivariable logistic regression analysis. Odds ratios (ORs) with corresponding 95% confidence intervals (CIs) and *p*-values are shown for specific treatment regimens associated with a positive QuantiFERON test. * rituximab or obinutuzumab.

	OR	95% CI	*p*-Value
Anti-CD20 *	0.89	0.43, 1.83	0.8
No anti-CD20			
Anti-CD20 *—bendamustine	0.63	0.27, 1.41	0.3
No Anti-CD20—bendamustine			
BTK inhibitor	2.07	0.76, 5.74	0.2
No BTK inhibitor			
Bcl-2 inhibitor (venetoclax)	1.18	0.39, 3.48	0.8
No Bcl-2 inhibitor (venetoclax)			
First line of treatment	0.49	0.23, 0.98	0.044
Multi-treated			

## Data Availability

Data available on request to the authors from the Hematology Unit due to privacy/ethical restrictions.

## Abbreviations

The following abbreviations are used in this manuscript:

CD20	Cluster of differentiation 20
BTK	Bruton tyrosine kinase
Bcl-2	B-cell lymphoma 2
CAR-T	Chimeric Antigen Receptor T cell
ECLIA	Electrochemiluminescence Immunoassay
AML	Acute myeloid leukemia
MDS	Myelodysplastic syndrome
PMF	Primary myelofibrosis
CLL	Chronic lymphocytic leukemia
SLL	Small lymphocytic lymphoma
NHL	Non-Hodgkin lymphoma
HL	Hodgkin lymphoma
GMT	Geometric mean titer
CI	Confidence interval
ORs	Odds ratios
Std Dev	Standard Deviation
Anti-S	Anti-spike
ND	Not determinable
CR	Complete remission
PR	Partial remission
O	Onset
SD	Stable disease
PD	Progressive disease
Ag	Antigen
BAUs	Binding Antibody Units
Pts	Patients
